# Exploring chitosan-shelled nanobubbles to improve HER2 + immunotherapy via dendritic cell targeting

**DOI:** 10.1007/s13346-022-01185-8

**Published:** 2022-06-07

**Authors:** Monica Argenziano, Sergio Occhipinti, Anna Scomparin, Costanza Angelini, Francesco Novelli, Marco Soster, Mirella Giovarelli, Roberta Cavalli

**Affiliations:** 1 Department of Drug Science and Technology, University of Turin, Via P. Giuria 9, 10125 Turin, Italy; 2 Department of Molecular Biotechnology and Health Science, University of Turin, Via Nizza 52, 10126 Turin, Italy

**Keywords:** Nanobubbles, Dendritic cells, Cancer immunotherapy, Targeted release, DNA vaccine

## Abstract

Immunotherapy is a valuable approach to cancer treatment as it is able to activate the immune system. However, the curative methods currently in clinical practice, including immune checkpoint inhibitors, present some limitations. Dendritic cell vaccination has been investigated as an immunotherapeutic strategy, and nanotechnology-based delivery systems have emerged as powerful tools for improving immunotherapy and vaccine development. A number of nanodelivery systems have therefore been proposed to promote cancer immunotherapy. This work aims to design a novel immunotherapy nanoplatform for the treatment of HER2 + breast cancer, and specially tailored chitosan-shelled nanobubbles (NBs) have been developed for the delivery of a DNA vaccine. The NBs have been functionalized with anti-CD1a antibodies to target dendritic cells (DCs). The NB formulations possess dimensions of approximately 300 nm and positive surface charge, and also show good physical stability up to 6 months under storage at 4 °C. In vitro characterization has confirmed that these NBs are capable of loading DNA with good encapsulation efficiency (82%). The antiCD1a-functionalized NBs are designed to target DCs, and demonstrated the ability to induce DC activation in both human and mouse cell models, and also elicited a specific immune response that was capable of slowing tumor growth in mice in vivo. These findings are the proof of concept that loading a tumor vaccine into DC-targeted chitosan nanobubbles may become an attractive nanotechnology approach for the future immunotherapeutic treatment of cancer.

**Graphical abstract:**

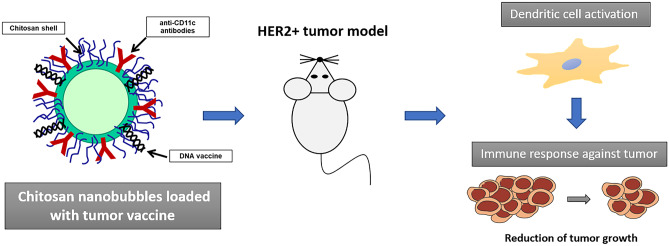

## Introduction

Immunotherapy has revolutionized our approach to cancer therapy [1]. To date, several immunotherapeutic agents have been approved by regulatory authorities for the treatment of many types of cancers. Immune checkpoint inhibitors, such as antibodies to cytotoxic T-lymphocyte-associated protein 4 (CTLA-4), programmed death-1 (PD-1), and programmed death-ligand 1 (PD-L1), have been in clinical use since 2011, and many other molecules with an inhibitory (e.g., TIM-3, LAG3) or co-stimulating effect (e.g., OX-40, CD137) are currently undergoing clinical trials [2]. Checkpoint immunomodulators have significantly improved outcomes for cancer patients, but evoke several adverse effects, including immune-related adverse events, which often limit their clinical use [3]. More recently, chimeric antigen receptor (CAR) T cell therapies have been approved for the treatment of advanced lymphocyte neoplasms [4], and other therapeutic strategies are in advanced clinical trials for solid tumor therapy. It is worth noting the remarkable efficacy of this new approach for the treatment of leukemia, myeloma, and lymphoma. However, the heterogeneity of the tumor microenvironment in solid cancer still remains a limitation difficult to overcome for this type of therapeutic agent [5]. The available therapies in clinical practice can be improved by targeting other immune cells that are involved in T cell activation, such as antigen presenting cells (APC) and natural killer (NK) cells [6]. Interestingly, a subcategory of APC, namely dendritic cells (DC), is particularly interesting for immunotherapy as they are capable of capturing and processing tumor antigens, allowing T cell recognition, and subsequent expansion, to fight cancer [7]. DC-based cancer vaccines have been explored as a promising therapeutic opportunity [8, 9], as cancer vaccination offers distinct advantages over standard therapies, such as specificity, lower toxicity, and long-term effects due to immunological memory [10]. The selection of the appropriate antigen plays a key role in the development of a DC-targeted vaccine. Interesting results have been obtained using recently discovered tumor neoantigens [11], and well-established tumor-associated antigens [12]. Taking into account the latter group, the HER2 oncogene is an excellent candidate for vaccine development [13, 14]. Its role has become increasingly evident and attracted much research with a number of formulations already in clinical trials [15, 16]. Furthermore, in order to properly expand the immune response against tumors, a vaccine must effectively target DCs, which play a critical role in inducing proper immune activation [10]. Nanotechnology approach has been explored to improve the efficacy of DC-based cancer immunotherapy. Nanoparticle vaccines can allow for increased antigen delivery to DCs as well as exhibiting a non-immunogenic nature and sustained antigen release capacity [17]. Indeed, nanomaterials can protect antigens and adjuvants from enzymatic degradation and enhance their cellular internalization into DCs, improving antigen immunogenicity and immune cell response [18, 19]. Several formulations, including poly(lactic acid) (PLA)/poly(lactic-co-glycolic acid) (PLGA) nanoparticles [20], liposomes [21], and solid lipid nanoparticles, have been proposed [22]. Furthermore, the decoration of nanoparticle surfaces with specific antibodies makes it possible to convey nanocarriers to the DCs, favoring the release of the tumor antigen. Recently, encouraging results were obtained exploiting the DC-targeted liposomal vaccine, L-BLP25, in a phase II clinical trial in patients with advanced non-small cell lung cancer (NSCLC) [23]. A number of research reports have been focused on the use of bubble liposomes (BL) for cancer vaccination [24]. Dendritic cell-based cancer immunotherapy has been proposed as an effective therapeutic strategy for metastatic melanoma and relapse because of prime tumor-specific cytotoxic T lymphocytes. DCs have been exposed to antigens in the presence of perfluoropropane-cored liposomes combined with ultrasound (US), and treated cells were used for prophylactic immunization of mice. Prophylactic immunization with BL/US-treated DC provided a four-fold decrease in the frequency of melanoma lung metastases [25].

Complexes with mannose-modified BLs and pDNA have also been used for immunization against cancer in mice in vivo, leading to a substantial increase in the secretion of cytokines TNFα, IFN-γ, IL-4, and IL-6, and also to enhanced cytotoxic T lymphocyte activity [26].

Combined treatments, using vaccines and anticancer agents, have also been studied. The antitumor effect of DNA vaccination against melanoma was enhanced using US-responsive mannose-modified bubble liposomes in combination with doxorubicin-encapsulated PEGylated liposomes. The effective cytotoxic activity of the T-lymphocytes that were stimulated by DNA vaccination was combined with the inhibition of tumor growth induced by doxorubicin [27].

A number of bubble systems have been investigated for gene delivery. Among them, polymer-shelled nanobubbles (NBs), spherical core/shell nanostructures filled with a gas or vaporizable compounds (i.e., perfluorocarbons), have shown a good capability to deliver nucleic acids [28–30]. NBs have gained increasing amounts of attention in the drug-delivery field because they can be loaded with drugs, gases, and genes in a good extent, providing controlled release [31–34]. They have therefore shown promise as innovative nanocarriers, as they also display better carrying capacity than microbubbles and good extravasation capability [35].

Furthermore, they are able to accumulate within tumor tissues via passive targeting, by exploiting the enhanced permeability and retention (EPR) effect [35], where they can be easily internalized by cancer cells. Moreover, an active targeting can be achieved by binding targeting ligands onto the NB surface [36]. In fact, the presence of the polymeric shell allows the NBs to be functionalized with specific targeted molecules. Interestingly, NBs can be visualized by US imaging, having good reflection capability, to follow their biodistribution and accumulation. In addition, it is possible to trigger the release of their payload in response to US application, obtaining site-specific delivery [37].

This work aims to design a new immunotherapeutic tool that exploits NB technology for the treatment of HER2 + breast cancer. For this purpose, we have developed innovative chitosan-shelled NBs that were loaded with a DNA vaccine and functionalized with an antibody to target DCs. The NBs were then characterized in vitro and in vivo to evaluate the cancer vaccination capability.

## Material and methods

### Materials

All reagents were of analytical grade and obtained from Sigma-Aldrich (St. Louis, MO, USA), unless otherwise specified. Medium molecular weight chitosan (degree of deacetylation 75–85%, 190–310 KDa), from Sigma-Aldrich, was used. Epikuron 200® was kindly provided by Cargill.

### DNA plasmids

A plasmid coding for GFP (pmaxGFP) and a plasmid pVAX1 coding for the extracellular and transmembrane domains of HER2, as previously described [13], were used for the transfection of DCs and in in vivo experiments.

### Preparation of nanobubble formulations

NBs were prepared as previously described, using perfluoropentane as the inner-core component and chitosan for the shell [32]. Briefly, an Epikuron 200 and palmitic acid (1% w/v) ethanol solution was added to perfluoropentane and ultrapure water. The mixture was then homogenized using an Ultra-Turrax® homogenizer (IKA, Konigswinter, Germany). Finally, a 2.7% w/v chitosan solution at pH 5.0 was added dropwise under mild stirring. Interestingly, to obtain the DNA-loaded NBs, the pmaxGFP plasmid was incorporated within the chitosan shell of pre-formed NBs via electrostatic interactions. The DNA-loaded chitosan NBs were then functionalized with either the anti-CD11c or anti-CD1a monoclonal antibodies (for the in vivo and in vitro experiments, respectively) via the amino-reductive method.

### Physico-chemical characterization of nanobubble formulations

Chitosan-shelled NBs, both blank and DNA-loaded (targeted and non-targeted), were characterized in vitro. The average diameter and polydispersity index of the NB formulations were determined by photon correlation spectroscopy, and the zeta potential was measured using electrophoretic mobility on a 90 Plus instrument (Brookhaven, NY). The analyses were carried out at a fixed angle of 90° and a temperature of 25 °C after the samples were diluted (1:30 v/v) with filtered water. For zeta potential determination, the diluted samples were placed in an electrophoretic cell to which a rounded 15 V/cm electric field was applied. The morphology of the NB formulations was evaluated by transmission electron microscopy (TEM), using a Philips CM10 instrument (Philips, Eindhoven, The Netherlands). Samples were dropped onto a Formvar-coated copper grid and air-dried prior to analysis.

### DNA complexation capacity of nanobubbles

The complexation of the pDNA with the chitosan-shelled NBs was evaluated using a gel retardation assay, via electrophoresis on an agarose gel.

DNA-loaded NBs were loaded into the agarose gel (1% w/v), stained with an ethidium bromide solution (0.5 μg/mL). The electrophoresis was run in TAE buffer (40 mM Tris base, 20 mM acetic acid, and 1 mM EDTA; pH 8.0) at 60 V for 1 h. A solution of pDNA (0.1 μg/μL) was used as a positive control. The banding pattern was visualized using an ultraviolet transilluminator and photographed with a Polaroid camera.

The amount of pDNA incorporated into the NBs was determined spectrophotometrically at 260 nm using a UV–visible spectrophotometer (DU 730, Beckman Coulter, Fullerton, CA) [38, 39]. The encapsulation efficiency was calculated by subtracting the amount of free pDNA from the initial amount added, according to the following equation:

$$Encapsulation \;efficiency = \frac{(Total \;pDNA-free \;pDNA)}{Total \;pDNA} \times 100$$

The loading capacity was determined over the freeze-dried NB samples, according to the equation:

$$Loading \;capacity=\frac{(Total\; pDNA-free\; pDNA)}{NB \;weight}\times 100$$

### Determination of NB physical stability over time

The physical stability of blank and DNA-loaded NBs (targeted and non-targeted) was evaluated over time. The average diameter, Z-potential, and morphology of the NB formulations, stored at 4 °C, were determined for up to 6 months.

### In vitro release of pDNA from nanobubbles

The in vitro release of pDNA from NBs was evaluated by incubating the non-targeted and targeted pDNA-loaded NBs (100 μg/ml pDNA concentration) in phosphate buffered saline (PBS) pH 7.4 at 37 °C ± 0.5 °C under magnetic stirring. At appropriate time intervals, an aliquot of the receiving medium was withdrawn and replaced with the same volume of fresh medium. The samples were then centrifuged (15,000 rpm for 15 min), and the amount of pDNA released into the supernatant was measured spectrophotometrically at 260 nm. The results were expressed as % of pDNA released over time, and represent the mean ± standard deviation (SD) of three independent experiments.

### Biocompatibility evaluation of nanobubble formulations

In order to evaluate the hemolytic activity of the NBs, different concentrations of the formulations were incubated with 1 mL of blood diluted with PBS pH 7.4 (1:10 v/v) at 37 °C for 90 min. After incubation, the samples were centrifuged (2000 rpm, 10 min) to separate the plasma, and the amount of hemoglobin released in the supernatant due to hemolysis was measured spectrophotometrically at 543 nm (Du 730 spectrophotometer, Beckman). The hemolytic activity was calculated with reference to completely hemolyzed samples (induced by the addition of Triton X-100 1% w/v to the blood, used as positive control) and a negative control (NaCl 0.9% w/v) [40].

Moreover, the cytotoxic activity of NBs was evaluated on a HaCat cell line. Cells were cultured at 0.5 × 10^6^ cells/mL in a 6-well-plate at 37 °C and 5% CO_2_ in Dulbecco’s modified Eagle’s medium (DMEM) supplemented with 10% fetal bovine serum (FBS), 2 mM glutamine, and 1% antibiotics (Gibco, Thermo Fisher Scientific, Waltham, MA, USA). After overnight incubation, the cells were treated for 24 h with the NBs at different concentrations. Cell viability was measured using the 3-(4,5-dimethylthiazol-2-yl)-2,5-diphenyltetrazoliumbromide (MTT) assay. The experiments were performed in triplicate and OD was measured at 570 nm using a microplate reader (VICTOR3TM, PerkinElmer, MA, USA).

### Generation and transfection of human dendritic cells

Human DCs were generated as previously described [13]. Human peripheral blood mononuclear cells (PBMCs) were isolated via centrifugation over a Ficoll gradient (Histopaque, Sigma) from the heparinized venous blood of healthy subjects, which as provided by the local Blood Bank (Turin, Italy). Monocytes were then obtained via MACS magnetic bead separation (Miltenyi Biotec) at a purity of > 93% CD14^+^. In order to generate hDCs, the monocytes were plated into six‐well culture plates (1.5 × 10^6^ cells/mL) (BD Falcon) in RPMI 1640 (Euroclone) supplemented with 10% heat‐inactivated certified FBS (HyClone) and incubated for 5 days, in the presence of GM‐CSF and IL‐4 (PeproTech, both 100 ng/mL). On the day of transfection, the hDCs were seeded with different amounts of GFP plasmid-loaded nanobubbles, both targeted with CD1a and non-targeted. After 24 h, the nanobubbles were removed and the medium was replaced with fresh RPMI + 10% certified FBS. Transfection efficiency was analyzed at 24 h via flow cytometry to evaluate the expression of GFP and DC viability. The specificity of the NBs that were targeted with CD1a for hDCs was evaluated via mixing, at a 1:1 ratio, 1 × 10^6^/ml DCs and 1 × 10^6^/ml PBMCs, obtained from the same healthy donor, in 24 well plates, and then transfecting them with targeted and non-targeted GFP-loaded NBs. Fluorescence was quantitated on a FACSCalibur flow cytometer that was equipped with CellQuest software (BD‐Biosciences). The PBMCs and DCs were distinguished according to their physical features. The amount of nanobubbles was evaluated using a NANOSIGHT (“Nanoparticle Tracking Analysis (NTA) Malvern”).

### Flow cytometry

In order to evaluate the ability of GFP-loaded nanobubbles to induce the maturation of the transfected hDCs, the expression of the CD86 and CD83 maturation markers [13] was evaluated via flow cytometry 24 h after the transfection of the hDCs with the targeted and non-targeted GFP-loaded nanobubbles. The following mAbs were used: αCD86-PE (clone IT2.2) and αCD83-PE (clone HB15e). Proper isotype‐matched control Abs (BioLegend) were used. Cells resuspended with FACS buffer (PBS supplemented with 0.2% BSA, 0.01% NaN_3_) were incubated with fluorochrome‐conjugated mAbs for 30 min at 4 °C, after non-specific sites were blocked with rabbit IgG (Sigma). Fluorescence was quantitated on a FACSCalibur flow cytometer (BD‐Biosciences). Cells were gated according to their light‐scatter properties to exclude cell debris.

### In vivo ability of NBs to migrate to lymph nodes

All animal studies were performed in accordance with EU and institutional guidelines and were approved by the Bioethics Committee for Animal Experimentation at the University of Turin, Turin, Italy, and by the Italian Ministry of Health (Prot. No. 1009/2015-PR). Intradermal injection has been performed as described with some modification [41].

BALB/c female mice (Charles River, 6–8 weeks of age) were anesthetized, and the back of the animal was shaved, to remove the hair, and was then swabbed with 70% ethanol. Mice received two intradermal injections (one on the left and one on the right side of the back), in the basal tail region, of either 20 μl of GFP-loaded CD11c-NBs or the same volume of naked-NBs, for use as a control. After 48 h, three mice per group were sacrificed and the inguinal lymph nodes were collected. The leukocytes extracted from the lymph nodes were stained with anti CD11c-PerCP mAb (Miltenyi Biotech) and analyzed by flow cytometry.

### Evaluation of the therapeutic efficacy of HER2-loaded NBs

Female BALB/c mice were challenged in the left flank with a lethal dose (3 × 10^5^) of human HER2-expressing D2F2/E2 mammary tumor cells [42]. Vaccination began when the tumor had reached a mean diameter of 2 mm. The mice received an intradermal injection of either 20 μl of the CD11c-targeted HER2-NBs, empty NBs, or the same volume of PBS, in the basal tail region, twice at a 14-day interval. Tumor growth was monitored every 5 days for 30 days after the first vaccination. Two perpendicular tumor diameters were measured with a caliper, and tumor volumes were calculated according to the formula: length × (width)^2^ × 0.5.

### ELISPOT assay

Splenocytes (spc) recovered at the necropsy of the mice that were vaccinated as described above were stimulated with D2F2/E2 cells at a 10:1 ratio for 48 h in a IFNγ ELISpot assay according to instructions provided by the manufacturer (BD Bioscience). In order to evaluate the CD8-restricted response, D2F2/E2 cells were incubated with MHC-I blocking antibody (BioLegend). Spots were counted with a computer-assisted image analysis system; the Transtec 1300 ELISPOT Reader (AMI Bioline). Specific spots were calculated by subtracting the spots that were produced by the spc in medium alone from the spots produced in presence of tumor cells.

### Statistical analysis

Statistical analyses were performed using Prism 5.0 GraphPad Software. Data are expressed as the median ± SEM. In vivo tumor growth is expressed as mean tumor volume.

## Results and discussion

Cancer vaccination is a powerful therapeutic strategy. This approach can benefit of targeted nanoformulations to specific cells. Dendritic cells (DCs) are the major determinants of vaccination due to their role in triggering antigen-specific immune responses. In recent years, the critical role of DCs in adaptive immunity has supported the development of DC-based vaccination strategies. A direct approach was attempted in which DCs are generated in vitro and loaded with tumor antigen prior to their autologous transfer to cancer patients. Aside from some successes in clinical trials, this procedure remains time-consuming and expensive. A simpler and more promising approach is based on the delivery of the antigen to DCs directly in vivo.

The rationale of this work was to improve DC-based cancer vaccination exploiting nanotechnology. Over the last decade, immunotherapy strategies involving nanotechnology-based approaches for the eradication of tumor cells to improve therapeutic outcomes have been intensely explored. A number of nanoparticles and nanomaterials have been studied for the targeted delivery of antigens to immune cells, increasing the effectiveness of immunotherapy [43–45]. In addition, another specific antitumor response can be further achieved by the combination of delivery systems with external stimuli, such as radiofrequency, magnetic fields, and ultrasound (US) [46].

In this context, microbubbles have been developed to be coupled with US to facilitate the localized release and uptake of immunotherapy molecules (i.e., antibodies, nucleic acids), promoting an immune response [47–49]. For example, the co-administration of a naked granulocyte–macrophage colony stimulating factor plasmid (pGM-CSF) combined with the checkpoint inhibitor, αPD-1, and targeted microbubbles coupled with US induced a remarkable antitumor immune effect in murine breast cancer model [50]. Nanobubbles are the second generation of bubbles, and can boast of higher stability and the capability to extravasate from blood circulation.

Interestingly, nanobubble technology has been proposed for diagnostic imaging, drug, and gene delivery. In particular, they represent a multifunctional nanoplatform able to store and protect high payload of nucleic acids and suitable for the co-delivery of different active molecules in the same nanostructure. Moreover, the polymeric shell can be easily functionalized with specific target ligands for an active targeted delivery [28].

Nanobubbles have recently been investigated as a means to activate immune systems [51–53]. For example, multiple combined anticancer treatments have been developed using nanobubbles loaded with sonosensitizers and immune checkpoint inhibitors. Antitumor immunity has been markedly improved with NB formulations via the maturation of dendritic cells and the activation of CD8 + cytotoxic T cells both in vitro and in vivo [51–53]. In this work, chitosan-shelled perfluoropentane-cored NBs that are functionalized with either anti-CD11c or anti-CD1a monoclonal antibodies have been purposely designed for targeted cancer vaccination. This formulation is referred to as “nanobubbles” for the sake of simplicity, but it would be more correct to use the term “nanodroplets,” as perfluoropentane is liquid at room temperature (boiling point of 29 °C). Anyway, nanodroplets underwent to the liquid-to-vapor transition in perfluoropentane upon the application of US, via a phenomenon called acoustic droplet vaporization [54, 55]. This phase change of perfluoropentane transforms nanodroplets into nanobubbles, very good reflector of US waves. It has therefore been demonstrated that they can be visualized by US imaging due to their echogenic properties [56].

Chitosan was selected for nanobubble shell due to its favorable features such as non-toxicity, biocompatibility, biodegradability, and positive charge. Chitosan nanoparticles were proven to be safe and therefore extensively investigated in nanobiomedical research as an effective drug delivery system. Moreover, chitosan has been reported to elicit significant adjuvant effects by promoting dendritic cell maturation by inducing type I interferons (IFNs) and enhance antigen-specific T helper 1 responses in a type I IFN receptor-dependent manner [57]. Previous studies have already reported that chitosan-based nanoparticles can be modified for in vivo delivery of different molecules directly to DCs. Chitosan nanoparticles have been employed to prime DCs by delivering whole-cell lysates from melanoma [58] or MUC1 peptide sequence as the immune-stimulatory component [59].

The main aim of the work is to investigate the capacity of this type of chitosan-shelled targeted nanobubbles to target and transfect dendritic cells.

Table 1 reports the average diameter, polydispersity index, and zeta potential of the nanobubble formulations, before and after loading with DNA.

**Table 1:** Physico-chemical characteristics of NB formulations

**Formulation**	**Average diameter** ** ± SD (nm)**	**PDI**	**Zeta potential** ** ± SD (mV)**
Blank chitosan-shelled NBs	392.6 ± 17.5	0.20 ± 0.02	+ 31.90 ± 2.3
pmaxGFP-loaded NBs	305.3 ± 23.5	0.21 ± 0.01	+ 15.37 ± 2.3
pmaxGFP-loaded NBs + αCD11c	311.6 ± 18.7	0.19 ± 0.03	+ 14.45 ± 1.5
pHER2-loaded NBs	303.2 ± 21.2	0.20 ± 0.02	+ 13.68 ± 1.9

**Fig. 1:**
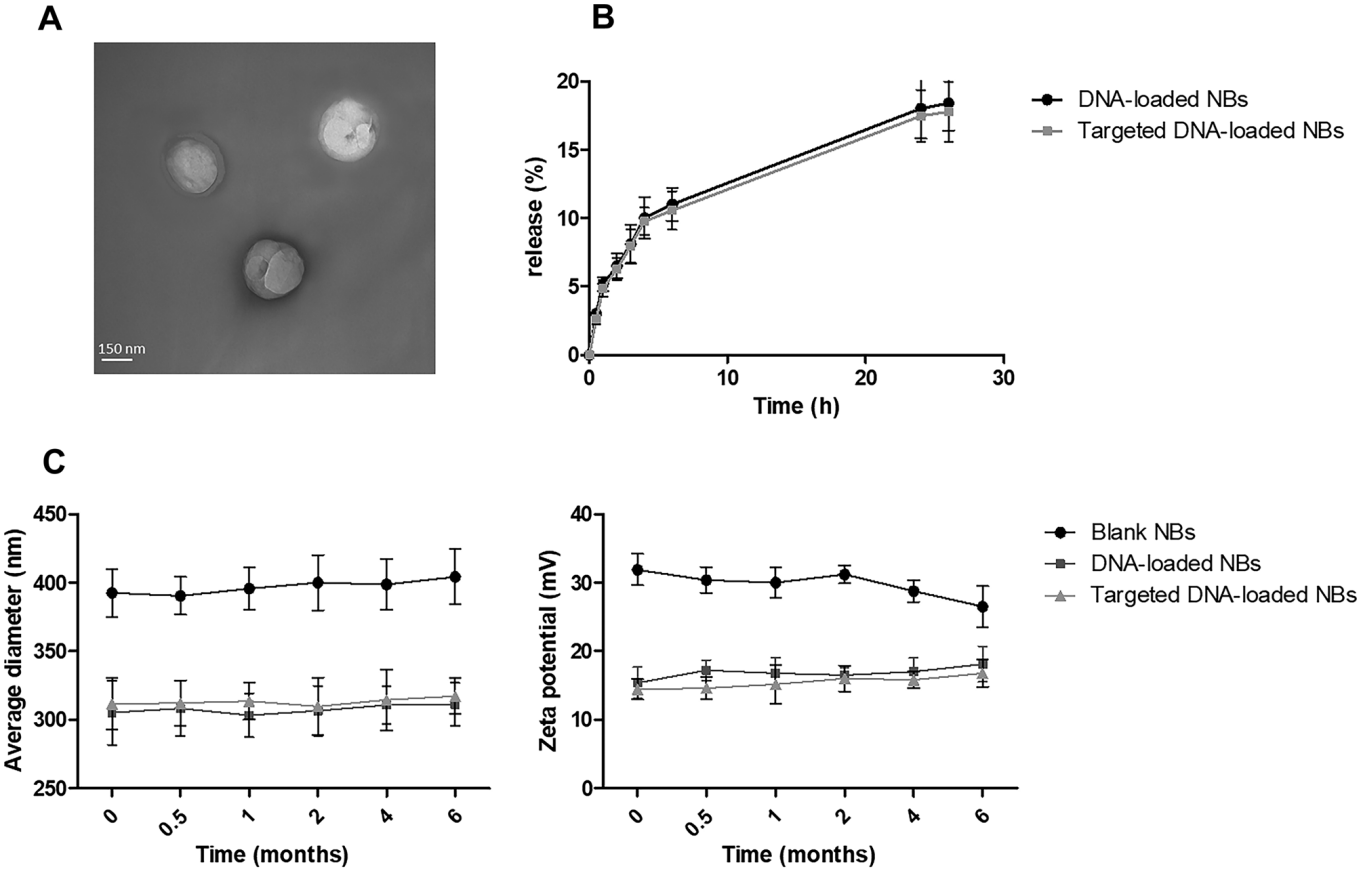
**A**) Transmission Electron Microscopy (TEM) image of DNA-loaded NBs. **B**) In vitro release kinetics of pDNA from targeted or non-targeted NBs. **C**) Evaluation of the NB physical stability over time

The targeted DNA-loaded NBs have sizes of about 300 nm and a well-defined core–shell structure, as demonstrated by TEM analysis (Fig. 1A).

Blank NBs have a positive surface charge, with a zeta potential of about + 30 mV. This indicates the presence of the positively charged chitosan chains on the NB surface.

Interestingly, a marked decrease in size and zeta potential values, of about 22.5 and 50%, respectively, was observed once the chitosan nanobubbles were incubated with plasmid DNA. These results may be related to the electrostatic interactions between the negative DNA phosphate groups and the positive amino groups of chitosan. This behavior has previously been observed with other DNA-loaded NB formulations, both chitosan and DEAE-dextran shelled ones [32, 60]. The polysaccharide shells showed a marked capability to incorporate and protect the DNA that was embedded in the polymer chains. The presence of DNA condensed the polymer chains due to electrostatic and hydrophobic interactions [61, 62], thus confirming plasmid localization and loading within the polymer shell. Here, pDNA encapsulation efficiency was about 82%, and the mass of the DNA was 3 × 10^−3^ μg/μm^2^.

pDNA complexation with chitosan-shelled NBs was confirmed using a gel retardation assay via electrophoresis in an agarose gel. The disappearance of the DNA band for DNA-loaded NBs was observed (data not shown). The in vitro pDNA release profiles of the targeted and non-targeted NB formulations are reported in Fig. 1B. pDNA was released from both NB formulations in a sustained manner. The prolonged and constant release kinetics indicated the incorporation of pDNA within the NB chitosan shell. NB physical stability was confirmed for up to 6 months using morphological analyses as well as size and Z-potential measurements over time. No significant changes in physico-chemical parameters were observed nor were aggregation phenomena (Fig. 1C). The absence of hemolytic activity and cytotoxicity in HaCat cells showed the biocompatibility of the NB formulations.

In order to validate the capacity of DNA-loaded NBs to target DCs, we simultaneously carried out experiments in vitro, in human cells and, in vivo, in mice.

**Fig. 2:**
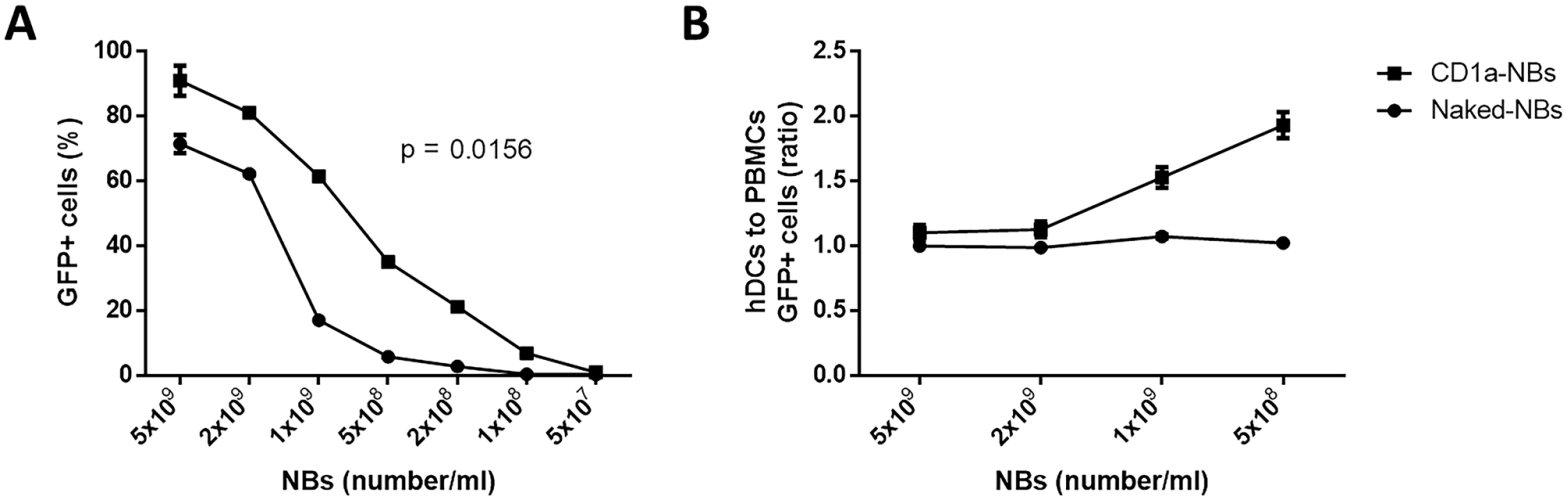
Transfection efficiency and specificity of NBs. **A**) Percentage of GFP + cells after incubation of hDCs with NBs targeted with CD1a (●) and naked-NBs (■) at different concentrations. **B**) Ratio of GFP + hDCs to GFP + PBMCs after incubation with naked (●) and NBs targeted with CD1a (■)

For this purpose, human DCs (hDCs) from CD14 + monocytes isolated from the venous blood of healthy subjects were generated. The hDCs were then incubated with different amounts of pmaxGFP-loaded NBs that had either previously been conjugated with an antibody specific for the hDCs marker, CD1a, or were left unconjugated and the transfection efficiency was analyzed by flow cytometry after 24 h. Interestingly, NBs targeted with CD1a showed more efficiency in transfecting DCs at all dilutions tested, compared to naked NBs (Fig. 2A).

In order to evaluate the specificity of the NBs targeted with CD1a to hDCs, hDCs and peripheral blood mononuclear cells (PBMCs) obtained from the same healthy donor were mixed at a 1:1 ratio. While naked chitosan-shelled NBs were incorporated by hDCs and PBMCs alike, the NBs targeted with CD1a showed a preferential transfection efficiency for hDC, compared to PBMCs (Fig. 2B).

Interestingly, targeted gene delivery exploiting antibody functionalization to direct nanobubbles to specific cells has previously been reported [63, 64]. In fact, the conjugation of antibodies onto nanocarrier surface can enhance their accumulation in specific tissues, avoiding unwanted biodistribution.

In this work, CD1a-functionalized NBs certainly showed high efficiency in transfecting hDCs with high selectivity for this cell type [65]. This finding suggests that NBs can play a crucial role in immunotherapy treatment.

Physiologically, once DCs encounter an antigen (Ag), they must migrate to lymph nodes where they present the Ag to Ag-specific T cells and induce T-cell activation and generation. That event requires a significant change in DC function and phenotype, which is also known as maturation. DC maturation is correlated with the upregulation of cell surface MHC molecules, co-stimulatory receptors, and relevant chemokine receptors that improve the ability of DCs to migrate to secondary lymphoid tissue [66]. In the absence of maturation stimuli, DCs fail to efficiently elicit a T cell response.

We therefore evaluated the ability of chitosan-shelled NBs to induce maturation in hDCs.

**Fig. 3:**
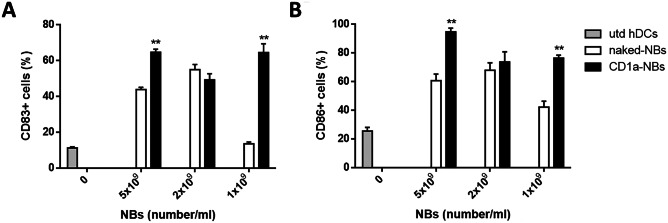
Expression of maturation markers. Expression of CD83 (**A**) and CD86 (**B**) on untreated hDCs (grey bar), hDCs transfected with decreasing amounts of naked NBs (white bar) and CD1a-NBs (black bar). ** *p* < 0.01

We evaluated the expression of the costimulatory and maturation marker molecules CD86 and CD83 using flow cytometry after the transfection of hDCs, and we observed that chitosan-shelled NBs are able to induce higher maturation-marker expression than untreated DCs. In addition, the increase is often higher with CD1a-NBs than with naked-NBs (Fig. 3).

Subsequently, we moved to the in vivo setting. As murine DCs (muDCs) are characterized by the expression of the surface marker CD11c, chitosan NBs were decorated with a monoclonal antibody that is specific for murine CD11c (CD11c-NBs).

**Fig. 4:**
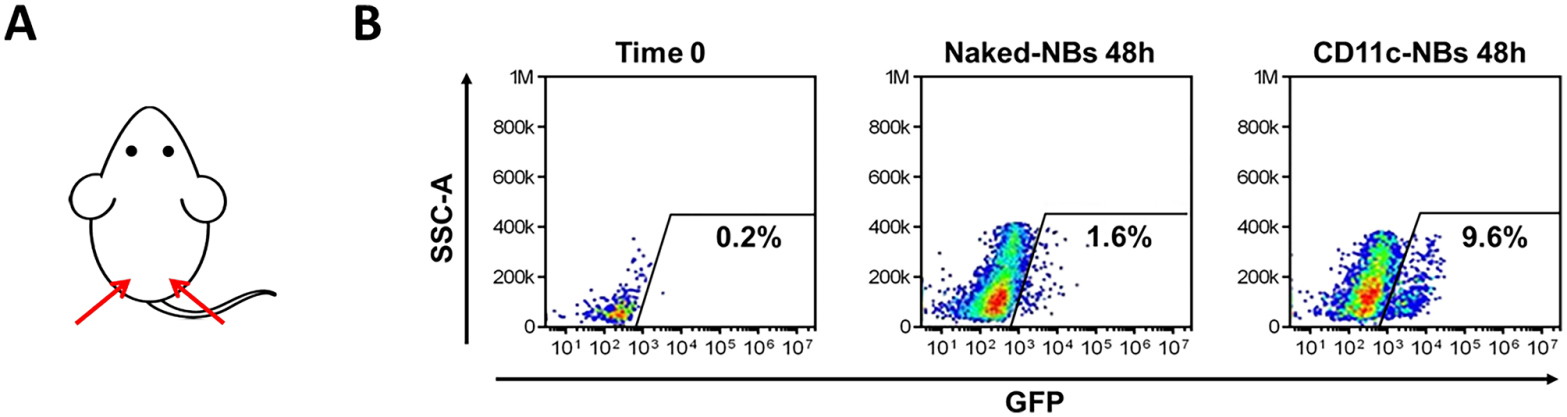
In vivo injection of GFP-loaded NBs. **A**) Injection site of NBs. **B**) Leucocytes extracted from lymph nodes at 48 h from injection were stained with anti-CD11c mAb and analyzed by flow cytometry. Percentages indicate GFP + cells gated on CD11c + cells. One representative mouse/group is shown

First, the ability of GFP-loaded CD11c-NBs to transfect in vivo muDCs and to elicit migration to draining lymph nodes was assessed. The mice either received two intradermal injections (one on the left and one on the right side of the back) of GFP-loaded CD11c-NBs or GFP-loaded naked-NBs, which were used as a control (Fig. 4A). Mice were sacrificed 48 h after injection and the inguinal lymph nodes were collected. The leucocytes extracted from the lymph nodes were stained with anti-CD11c mAb and analyzed by flow cytometry. The results showed that higher amounts of CD11c cells were detected in the CD11c-NB-treated mice than in the control, suggesting a migration of muDCs was induced by NB treatment (Fig. 4B). These findings might pave the way for the development of a new therapeutic strategy that might permit cooperation between nanomedicine and immunotherapy.

In order to demonstrate the ability of targeted chitosan-shelled NBs to elicit an antitumor immune response, BALB/c mice were subcutaneously challenged with D2F2/E2 cells [42], which are a murine cell line that expresses human ErbB-2 (HER2), which is an oncogene that is overexpressed in many kinds of human tumors [67].

When the mice displayed established palpable tumors, they received two rounds of an intradermal injection of CD11c-NBs loaded with a plasmid coding for HER2 (HER2-NBs), or with an empty vector (empty-NBs), or the same volume of PBS.

**Fig. 5:**
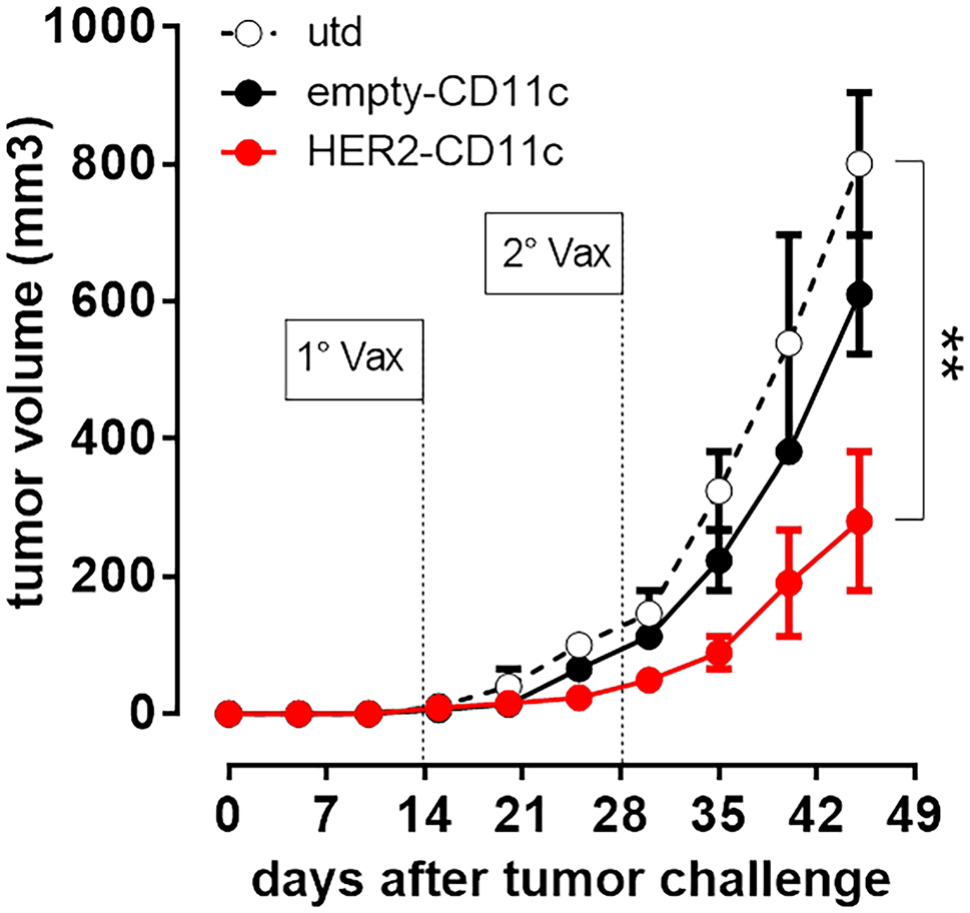
Anti-tumor vaccination with HER2-NBs. BALB/c mice were challenged subcutaneously in the left flank with 3 × 10.^5^ D2F2/E2 cells. When the tumor had reached a mean diameter of 2 mm, mice received an intradermal injection of either 20 µl of HER2-NBs (●), empty-NBs (●) or the same volume of PBS (○), twice at a 14-day interval. Tumor growth was monitored with calipers every 5 days for 30 days. ** *p* < 0.01 to utd

Tumor growth was monitored by caliper every 5 days for 30 days, after which the mice were sacrificed. Mice treated with HER2-NBs displayed a delay in tumor growth compared to untreated mice and the mice injected with empty-NBs (Fig. 5).

**Fig. 6:**
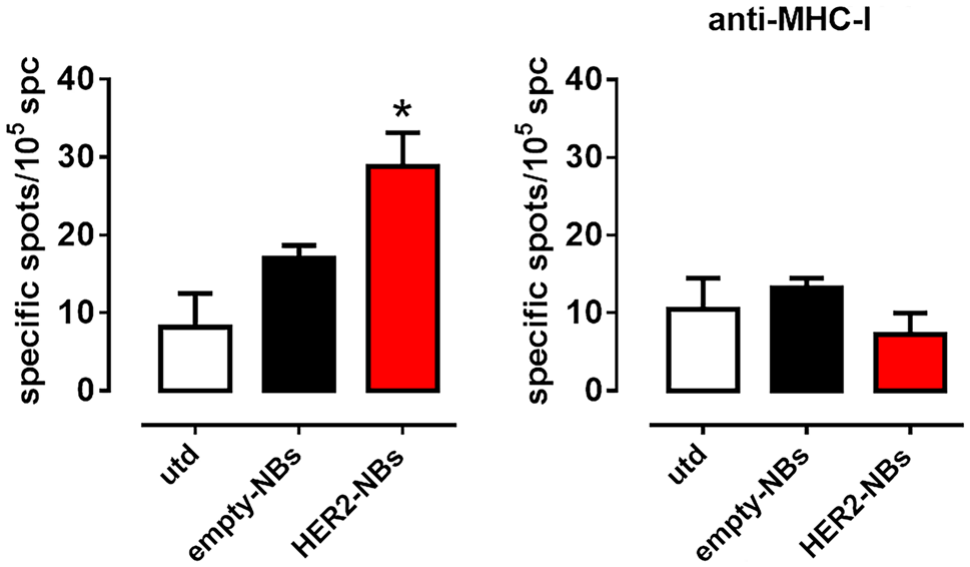
HER2-NBs elicit an anti-HER2 T cell IFNγ response. Spc were cultured with D2F2/E2 cells at a 10:1 ratio for 48 h in an IFNγ ELISpot assay. In order to evaluate the CD8-restricted response, D2F2/E2 were incubated with the MHC-I blocking antibody. Specific spots were calculated by subtracting the spots produced by the spc in medium alone from the spots produced in the presence of tumor cells. * *p* < 0.05 to utd

The specificity of the T-cell response against human HER2 was assessed using an IFN-γ ELISPOT assay, and the spc recovered at necropsy were stimulated with D2F2/E2 cells. Compared to the control group, the spc from mice vaccinated with HER2-NBs secreted a larger amount of IFN-γ against HER2 + tumor cells (Fig. 6A) and these cells were mainly CD8 T cells (Fig. 6B).

## Conclusions

In this study, chitosan-shelled NBs loaded with DNA vaccine and targeted to DCs have been successfully developed for the treatment of HER2 + breast cancer. This type of NBs was able to load DNA with good encapsulation efficiency and release it with prolonged and controlled release kinetics. They displayed the capability to transfect with high selectivity the DCs and induce their activation both in human and mouse cell lines. Additionally, intradermal injection of targeted DNA-loaded NBs delayed tumor growth in vivo in HER2 + breast cancer mouse model by eliciting a specific immune response. Interestingly, in the future, the visualization of nanobubble distribution by US imaging may be feasible as well as the co-loading of tumor vaccine with drugs for combined anticancer treatments. DC-targeted chitosan nanobubbles loaded with a tumor vaccine may become an attractive nanoplatform with promising features for a future clinical translation in immunotherapy.

## Data Availability

The datasets generated during and/or analyzed during the current study are available from the corresponding author on reasonable request.
